# Protein corona: implications for nanoparticle interactions with pulmonary cells

**DOI:** 10.1186/s12989-017-0223-3

**Published:** 2017-10-30

**Authors:** Nagarjun V. Konduru, Ramon M. Molina, Archana Swami, Flavia Damiani, Georgios Pyrgiotakis, Paulo Lin, Patrizia Andreozzi, Thomas C. Donaghey, Philip Demokritou, Silke Krol, Wolfgang Kreyling, Joseph D. Brain

**Affiliations:** 1 Department of Environmental Health, Molecular and Integrative Physiological Sciences Program, Harvard T.H. Chan School of Public Health, 665 Huntington Avenue, Boston, MA 02115 USA; 2 Center for Nanotechnology and Nanotoxicology, Harvard T.H. Chan School of Public Health, 665 Huntington Avenue, Boston, MA 02115 USA; 3 CIC biomaGUNE Soft Matter Nanotechnology Group, Paseo de Miramón, 182, 20014 San Sebastian-Donostia, Guipuzcoa Spain; 4 Fondazione I.R.C.C.S. Istituto Neurologico Carlo Besta, Via Amadeo 42, 20133 Milan, Italy; 5 I.R.C.C.S. Istituto Tumori Giovanni Paolo II, Viale O. Flacco 65, 70124 Bari, Italy; 6 Institute of Epidemiology 2, Helmholtz Zentrum München - German Research Center for Environmental Health, Ingolstädter Landstraße 1, 85764 Oberschleißheim, Germany; 7 IFOM, via Adamello 16, 20139 Milano, Italy

**Keywords:** Engineered nanoparticles, Biokinetics, Nanotoxicity, Protein corona, Lung macrophage

## Abstract

**Background:**

We previously showed that cerium oxide (CeO_2_), barium sulfate (BaSO_4_) and zinc oxide (ZnO) nanoparticles (NPs) exhibited different lung toxicity and pulmonary clearance in rats. We hypothesize that these NPs acquire coronas with different protein compositions that may influence their clearance from the lungs.

**Methods:**

CeO_2_, silica-coated CeO_2_, BaSO_4_, and ZnO NPs were incubated in rat lung lining fluid in vitro. Then, gel electrophoresis followed by quantitative mass spectrometry was used to characterize the adsorbed proteins stripped from these NPs. We also measured uptake of instilled NPs by alveolar macrophages (AMs) in rat lungs using electron microscopy. Finally, we tested whether coating of gold NPs with albumin would alter their lung clearance in rats.

**Results:**

We found that the amounts of nine proteins in the coronas formed on the four NPs varied significantly. The amounts of albumin, transferrin and α-1 antitrypsin were greater in the coronas of BaSO_4_ and ZnO than that of the two CeO_2_ NPs. The uptake of BaSO_4_ in AMs was less than CeO_2_ and silica-coated CeO_2_ NPs. No identifiable ZnO NPs were observed in AMs. Gold NPs coated with albumin or citrate instilled into the lungs of rats acquired the similar protein coronas and were cleared from the lungs to the same extent.

**Conclusions:**

We show that different NPs variably adsorb proteins from the lung lining fluid. The amount of albumin in the NP corona varies as does NP uptake by AMs. However, albumin coating does not affect the translocation of gold NPs across the air-blood barrier. A more extensive database of corona composition of a diverse NP library will develop a platform to help predict the effects and biokinetics of inhaled NPs.

## Background

Human exposures to industrially-relevant nanoparticles (NPs) are mainly by inhalation and are increasing in occupational and environmental settings and from releases from various nano-enabled products across their lifecycle [1–4]. Airborne NPs deposited in respiratory tract airways are cleared by the mucociliary apparatus; others reach the alveolar regions of the lung where they interact with the alveolar lining fluid. A very few may pass through the layer of pneumocytes and translocate to blood where they may reach other organs [5, 6]. Most particles deposited in the distal lung are ingested by lung macrophages and ultimately dissolve [7, 8].

In alveolar spaces, corona formation takes place in the alveolar lining fluid. It consists of plasma proteins, a surface-active phospholipid (PL)-protein mixture, also known as pulmonary surfactant, and a thin layer of aqueous hypophase especially in the alveolar “corners” [9]. Pulmonary surfactant is composed of 85–90% (*w*/w) PLs and 10% (w/w) of surfactant-specific proteins, such as the hydrophilic surfactant proteins A (SP-A) and D (SP-D). The hydrophobic surfactant proteins B (SP-B) and C (SP-C) are present to a lesser extent (<5% of the surfactant proteins, w/w) [10]. Studies have shown a pivotal role for surfactant proteins in lung host defense. SP-A and SP-D opsonize inhaled microbes, allergens, and other foreign bodies such as NPs to varying degrees, and promote their recognition, ingestion and dissolution by resident alveolar macrophages (AMs) and other leukocytes [11, 12].

NP surface charge, covalent/coordinate bonding and hydrogen bonding propensity are important NP surface properties that may influence their adsorption of lipids and proteins [13, 14]. The effects of hydrophobicity and surface charge of engineered nanoparticles (ENPs) on their binding to hydrophobic, positively-charged proteins SP-B and SP-C have been reported [15, 16]. It was found that anionic nanoparticles selectively adsorbed SP-B_1–25_, but not SP-C. Recent studies showed that inhaled diesel exhaust NPs exhibit a corona of SP-A, SP-D, and albumin [17]. The role of albumin in the translocation of intravenously injected gold NPs (AuNPs) to different organs has been described [18]. However, albumin’s influence on translocation across the air-blood barrier is unknown. Schleh et al. studied the effect of SP-D on the translocation of Au NPs (20 nm) from the lungs to the circulation [19]. They reported that SP-D had only a minor effect on early Au NP translocation.

Adsorption of biomolecules on to the NP surface occurs rapidly and results in the formation of a phospholipid-protein ‘corona’ on the NP surface [20]. The determinants of corona composition are underappreciated and poorly characterized, but are critical in determining the subsequent fate and effects of NPs in both in vitro and in vivo systems. It is reasonable to assume that the interactions of NPs with lung cells occur with the NP-protein phospholipid complex and not with a pristine NP surface. The protein corona thus formed can significantly affect the manner in which lung cells interact with, recognize, and process NPs [20]. The influence of the NP corona on the subsequent responses of the lungs to NPs needs to be better characterized.

Translocation of gold, silver, TiO_2_, polystyrene, and carbon-based particles across the air-blood barrier into the circulation and extrapulmonary organs, although small and poorly understood, has been described in a review [21]. We have performed pulmonary toxicological and biokinetic studies of cerium oxide (CeO_2_), barium sulfate (BaSO_4_) and zinc oxide (ZnO) NPs [22–24]. We also studied the consequences of a surface-modified CeO_2_ with a nanothin amorphous SiO_2_ coating (Si-CeO_2_) on clearance of the core cerium NPs [23]. In these studies, we explored whether radiolabeled ^141^CeO_2_, Si-^141^CeO_2,_^131^BaSO_4_ and ^65^ZnO NPs of similar size differ in lung clearance after intratracheal (IT) instillation. We showed that the pulmonary clearance was ^65^Zn > ^131^Ba > ^141^Ce (Si-CeO_2_ > ^141^Ce (CeO_2_) [22–24]. Of the four NPs examined, ^65^Zn cleared the lung fastest most likely due to high dissolution of ^65^ZnO NPs [25]. Among the other three NPs, ^131^Ba cleared the lung fastest (84% of the instilled dose cleared the lung by day 28). ^141^Ce from silica-coated ^141^CeO_2_ was cleared from the lungs relatively faster than from uncoated ^141^CeO_2_ [23]. These differences may be influenced by how these NPs interact with the lung lining fluid resulting in corona formation which in turn determine the NP fate and biological effects.

As the corona may also modulate the overall NP biokinetics and biological effects, we now further characterize the physicochemical properties of CeO_2_, Si-CeO_2_, BaSO_4_ and ZnO NPs. These properties include surface charge, agglomeration in various fluids, and the composition of the protein corona formed when incubated in concentrated bronchoalveolar lavage fluid (BALf). Finally, we tested the role of albumin, the major protein in the lung lining fluid, on the clearance of instilled gold NPs in rats and on the acquisition of protein corona when incubated in BALf.

## Results

### Nanoparticle physicochemical characterization

**Fig. 1:**
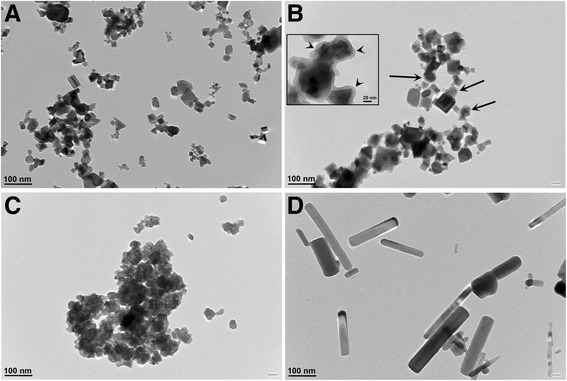
Transmission electron micrographs (TEM) of nanoparticle suspensions in DI water. **a** CeO_2_. **b** Si-CeO_2_. Nanothin coatings of amorphous silica are shown (arrows). A higher magnification of additional Si-CeO_2_ NPs are shown in the inset. The silica coating appears as less electron dense coating (arrowheads) surrounding the core CeO_2_ NPs. **c** BaSO_4_. **d** ZnO nanorods

The synthesis of CeO_2,_ Si-CeO_2_ and ZnO NPs used in this study is outlined in detail in our previous publications and summarized in the methods section. BaSO_4_ NPs (NM-220) were obtained from BASF SE (Ludwigshafen, Germany). Transmission electron micrographs of NPs suspended in deionized (DI) water are shown in Fig. 1. Table 1 lists selected physicochemical characteristics of the NPs. The primary NP sizes were similar (29 to 36 nm). The surface areas were identical for the two CeO_2_ NPs, while the BaSO_4_ and ZnO NPs had higher specific surface areas. The hydrodynamic diameter of NP suspensions in DI and zeta potentials were measured before incubation in harvested cell-free rat BALf for 30 min at 37 °C by dynamic light scattering (DLS) using a Malvern ZetaSizer Nano (Westborough, MA). The NPs were incubated in BALf to examine the formation of the protein corona and its influence on NP agglomerate size and zeta potential. The multiple washing steps removed unbound and loosely bound proteins from the NPs. The zeta potentials of CeO_2_ and ZnO NPs changed from positive to negative, and the hydrodynamic diameters of all NPs increased after incubation in BALf. After the acquisition of NP coronas, the conductance of the NP suspensions in DI water increased dramatically (Table 1).

**Table 1:** Characteristics of nanoparticles

	CeO_2_	Si-CeO_2_	BaSO_4_	ZnO
SSA (m^2^/g)	28.0	27.8	34.6	41.0
D_xrd_ (nm)	32.9	32.6	36.0	29.0
ρ (g/cm^2^)	7.65	~7.20	4.50	5.61
NPs in DPBS				
D_H_ (nm)	1254 ± 110	378 ± 13	191 ± 13	2524 ± 343
PdI	0.35 ± 0.04	0.30 ± 0.05	0.38 ± 0.03	0.29 ± 0.03
Conductance (mS/cm)	13.4 ± 0.46	14.3 ± 0.49	14.2 ± 0.64	13.7 ± 0.17
ζ (mV)	−21.4 ± 0.46	−23.5 ± 0.55	−30.7 ± 1.92	−28.2 ± 5.26
NPs without corona in DI water				
D_H_ (nm)	136 ± 1	208 ± 3	154 ± 21	221 ± 3
PdI	0.29 ± 0.03	0.29 ± 0.02	0.29 ± 0.005	0.23 ± 0.02
Conductance (mS/cm)	0.016 ± 0.001	0.018 ± 0.0002	0.011 ± 0.001	0.04 ± 0.007
ζ(mV)	34.5	−26.8	−18.7	23.0
NPs with coronas (post-30 min incubation in BALf) in DI water				
D_H_ (nm)	1513 ± 17	460 ± 12	595 ± 17	2189 ± 64
PdI	0.49 ± 0.02	0.35 ± 0.02	0.48 ± 0.02	0.30 ± 0.05
Conductance (mS/cm)	14.7 ± 0.87	15.1 ± 0.76	14.8 ± 0.81	14.6 ± 0.23
ζ (mV)	−19.0 ± 0.91	−19.2 ± 0.66	−15.2 ± 1.76	−17.7 ± 2.56

*SSA* specific surface area measured with BET analysis

*D*_*xrd*_ primary particle size based on X-ray diffraction

*ρ* density, Si-CeO_2_ (based on 91:9 ratio of CeO_2_:SiO_2_)

*D*_*H*_ hydrodynamic diameter by DLS

*PdI* polydispersity index

*ζ* zeta potential by DLS

*DI water* deionized water

*DPBS* Dulbecco’s phosphate buffered saline

*BALf* bronchoalveolar lavage fluid

### Protein corona characterization

**Fig. 2:**
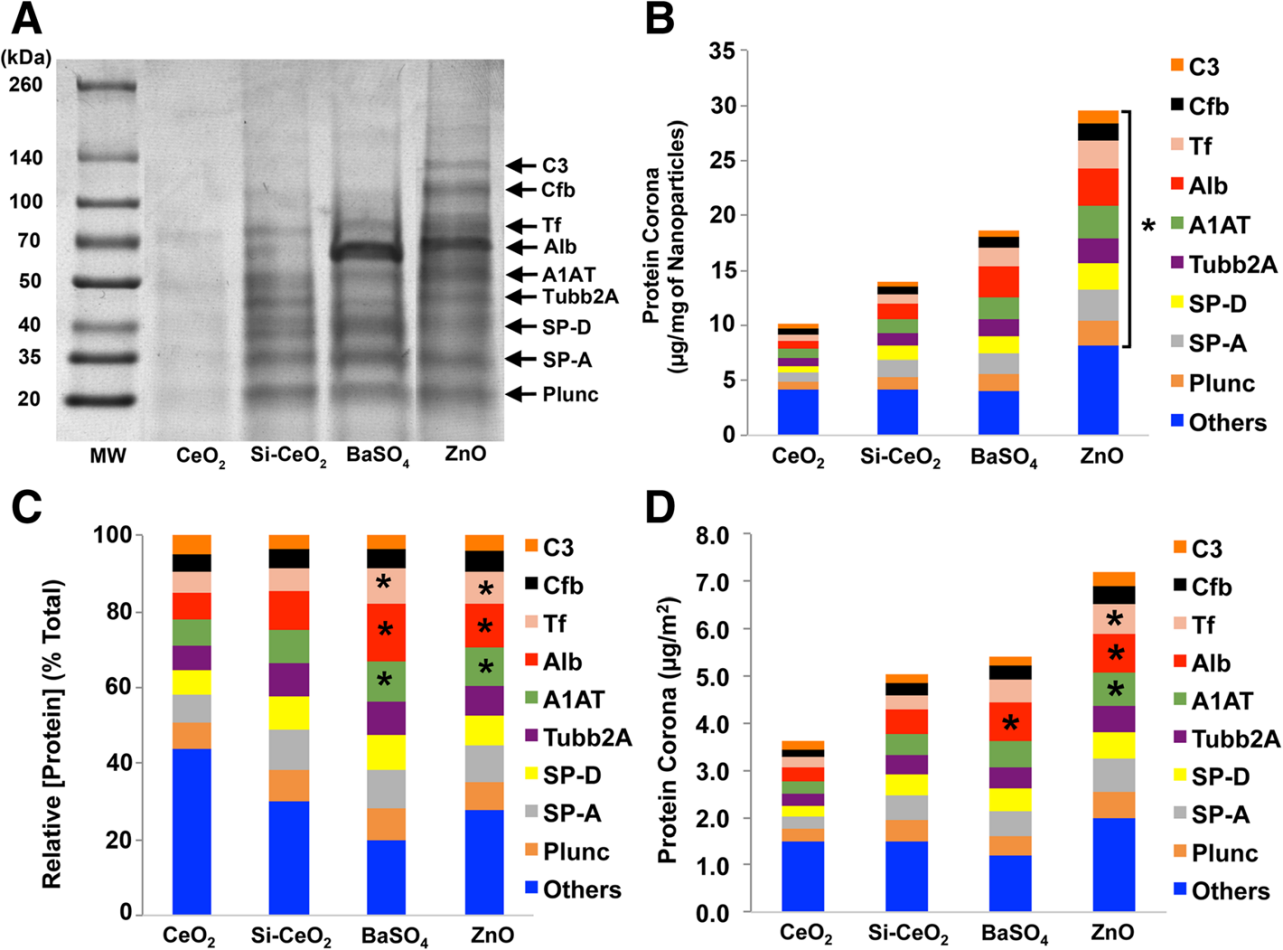
Analysis of NP-bound proteins by 1D gel electrophoresis and mass spectrometry. **a** The molecular weights (kDa) of reference proteins are shown in lane MW. A representative gel from one experiment is shown. NP-corona components identified by LC-MS are indicated on right. **b** LC-MS profiles of the most abundant BAL proteins and influence of NP type on corona profile. The nine most abundant proteins are shown. The total proteins per mg NPs were in the order of CeO_2_ = Si-CeO_2_ = BaSO_4_ < ZnO. (* *P* < 0.05, significant variations in each protein among 4 NP types). **c** When expressed as % of total bound protein, transferrin, albumin and α-1-antitrypsin were greater in the corona of ZnO and BaSO_4_ than of both CeO_2_ NPs. (* *P* < 0.05, significant variations in each protein among 4 NP types). **d** When expressed as bound proteins per surface area of NPs, transferrin and α-1-antitrypsin were greater in the corona of ZnO than of CeO_2_ NPs. Bound albumin was greater in the coronas of ZnO and BaSO_4_ than of both CeO_2_ NPs. Data are mean ± SD, *n* = 3 rats per NP)

We analyzed the composition of the acquired NP protein corona using methods described previously [26]. We performed 1-D SDS-PAGE and Coomassie staining of total proteins detached from the NPs to visualize the corona components on gel bands. The proteins in the excised gel bands were identified in an LTQ Orbitrap Velos Pro ion-trap mass spectrometer. The stained SDS-PAGE gel and the relative amounts of identified proteins after NPs were incubated with BALf are shown in Fig. 2. We found that the protein composition of the NP coronas were different. The total amount of protein bound per unit mass (μg/mg NPs) was in the order of CeO_2_ < Si-CeO_2_ < BaSO_4_ < ZnO (Fig. 2b). The amounts of individual proteins in the corona are shown in Table 2. When expressed as % of total bound protein, albumin, transferrin and α-1-antitrypsin were relatively higher in the corona of BaSO_4_ and ZnO than that of CeO_2_ and Si-CeO_2_ NPs (Fig. 2c). When expressed as bound proteins per surface area (μg/m^2^), ZnO NPs acquired greater amounts of transferrin, albumin and α-1-antitrypsin and BaSO_4_ NPs acquired greater amounts of albumin than did both CeO_2_ and Si-CeO_2_ NPs (Fig. 2d).

**Table 2:** Quantification of corona proteins on nanoparticles (A. μg/mg NPs; B. µg/m^2^ NPs)

	CeO_2_	Si-CeO_2_	BaSO_4_	ZnO
A.				
C3	0.46 ± 0.19	0.47 ± 0.12*	0.63 ± 0.27	1.21 ± 0.37*
Cfb	0.48 ± 0.25^#^	0.71 ± 0.26*	0.98 ± 0.31	1.54 ± 0.35*^#^
Tf	0.60 ± 0.39^#^	0.84 ± 0.23*	1.67 ± 0.36	2.57 ± 0.95*^#^
Albumin	0.78 ± 0.54*^#^	1.47 ± 0.66^@^	2.79 ± 0.27*	3.38 ± 0.79^#@^
A1AT	0.74 ± 0.45*	1.21 ± 0.17^#^	1.98 ± 0.49	2.91 ± 0.72*^#^
Tubb2A	0.72 ± 0.49*	1.20 ± 0.26^#^	1.57 ± 0.14	2.29 ± 0.52*^#^
SP-D	0.65 ± 0.40*	1.23 ± 0.34	1.61 ± 0.37	2.35 ± 0.63*
SP-A	0.80 ± 0.46*	1.48 ± 0.31	1.82 ± 0.29	2.89 ± 1.24*
PLUNC	0.72 ± 0.38*	1.17 ± 0.20	1.51 ± 0.28	2.23 ± 0.66*
Others	4.17 ± 1.73	4.20 ± 0.94	4.11 ± 3.37	8.18 ± 2.52
B.				
C3	0.16 ± 0.07	0.17 ± 0.04	0.18 ± 0.08	0.29 ± 0.09
Cfb	0.17 ± 0.09	0.25 ± 0.09	0.28 ± 0.09	0.38 ± 0.08
Tf	0.22 ± 0.14*	0.30 ± 0.08	0.48 ± 0.10	0.63 ± 0.23*
Albumin	0.28 ± 0.19*^#^	0.53 ± 0.24	0.81 ± 0.08*	0.82 ± 0.19^#^
A1AT	0.26 ± 0.16*	0.44 ± 0.06	0.57 ± 0.14	0.71 ± 0.18*
Tubb2A	0.26 ± 0.18^@^	0.43 ± 0.09	0.45 ± 0.04	0.56 ± 0.13^@^
SP-D	0.23± 0.14^@^	0.44 ± 0.12	0.47 ± 0.11	0.57 ± 0.15^@^
SP-A	0.29 ± 0.16	0.53 ± 0.11	0.53 ± 0.08	0.71 ± 0.30
PLUNC	0.26 ± 0.14	0.42 ± 0.07	0.44 ± 0.08	0.54 ± 0.16
Others	1.49 ± 0.62	1.51 ± 0.34	1.19 ± 0.97	2.00 ± 0.61

Data are mean ± SD of triplicate analyses using pooled BALf from 3 rats per NP

The pair of NPs with *, ^#^ or ^@^ are significantly different from each other, *P* < 0.05, Tukey adjustment for multiple comparisons

*C3* complement component 3, *Cfb* complement factor B, *Tf* transferrin, *A1AT* α1-antitrypsin, *Tubb2A* Tubulin Beta 2A Class IIa, *SP-D* Surfactant protein-D, *SP-A* Surfactant protein-A, *PLUNC* Palate, lung, nasal epithelium clone protein

### Nanoparticle uptake by alveolar macrophages

**Fig. 3:**
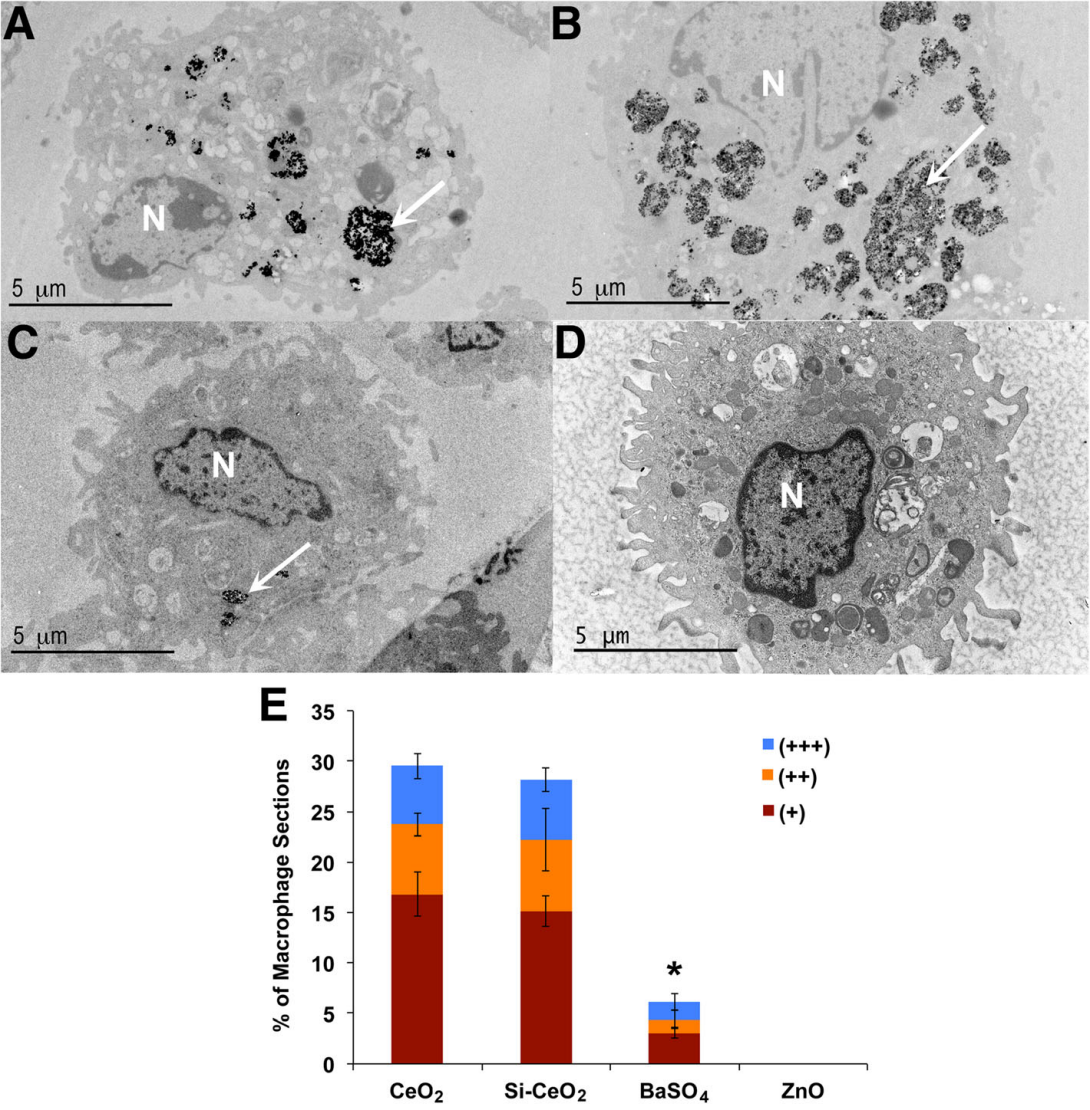
Transmission electron micrographs of lavaged cells at 24 h from rats instilled with NPs at a dose of 1 mg/kg body weight. Macrophage uptake was scored as +, ++, or +++ when 1–2, 3–4 or ≥5 particle-containing phagosomes were observed in macrophages, respectively. **a** CeO_2_, **b** Si-CeO_2_, **c** BaSO_4_ and **d**. ZnO. No identifiable ZnO NPs were observed in lavaged cells from ZnO-instilled rats. **e** Morphometric analysis of macrophage uptake of NPs (*n* = 248, CeO_2_, *n* = 273, Si-CeO_2_, *n* = 295, BaSO_4_, *n* = 250, ZnO). A significantly smaller fraction of lavaged macrophages showed uptake of BaSO_4_ NPs compared with CeO_2_ and Si-CeO_2_ NPs. Data are mean ± SE of % of macrophages (*n* = 3 rats per NP). * *P* < 0.05, BaSO_4_ vs. CeO_2_ and Si-CeO_2_ NPs

To explore if AM uptake of CeO_2_, Si-CeO_2_, BaSO_4_ and ZnO NPs in vivo were different, we instilled rats intratracheally with NP suspensions at a dose of 1 mg/kg body weight. Then, we measured the degree of phagocytosis of these NPs by AMs recovered from BALf at 24 h. Using transmission electron microscopy (TEM), we found that the % of sections of AMs with internalized BaSO_4_ NPs was less than CeO_2_ and Si-CeO_2_ (Fig. 3). No identifiable ZnO NPs were observed. An additional TEM examination at 1-h post-instillation also did not show ZnO NPs in lavaged cells (data not shown).

### Role of albumin in translocation and AM uptake of gold nanoparticles

**Fig. 4:**
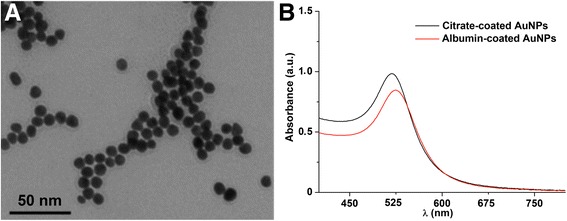
**a** Transmission electron micrograph of citrate-coated Au NPs. **b** UV-vis spectra of citrate-coated Au NPs in water (black curve) and albumin-coated Au NPs in PBS (red curve). The Au NPs show a red-shift in the peak absorbance wavelength after albumin coating

Our results showed that levels of some proteins in the NP corona, such as transferrin, albumin and A1AT (α1-antitrypsin), varied among the 4 NPs. As shown in Fig. 2c, BaSO_4_ and ZnO NPs had the highest level of albumin in their corona. Therefore, we tested the influence of albumin coating on the fate of instilled gold (Au) NPs since it was the major protein observed in NP coronas. We coated Au NPs (core size 18 ± 2 nm) with either albumin or citrate and studied their translocation across the air-blood barrier. Fig. 4a shows a TEM image of citrate-coated Au NPs and Table 3 shows hydrodynamic diameter and zeta potential of citrate- or human serum albumin-coated Au NPs in deionized water and in BALf. The hydrodynamic diameter in deionized water was 25 ± 0.2 nm (citrate-coated Au NPs) and 53 ± 0.1 nm (albumin-coated Au NPs). When incubated in BALf, the hydrodynamic diameter of citrate- and albumin-coated Au NPs changed from 25 to 275 nm and 53 to 115 nm, respectively. There was also a change in negative zeta potential value of the albumin-coated Au NPs (−14 to −56 mV). Fig. 4b shows the absorption spectra of citrate-coated Au NPs in water (black curve) and of albumin-coated Au NPs in PBS.

**Table 3:** Characteristics of Au nanoparticles

	Citrate-coated Au NPs	Albumin-coated Au NPs		
NPs in DI water				
D_H_ (nm)	25 ± 0.2	53 ± 0.1		
PdI	0.51 ± 0.004	0.36 ± 0.002		
Conductance (mS/cm)	0.077 ± 0.006	0.064 ± 0.007		
ζ(mV)	−46.17 ± 2.17	−14.25 ± 0.35		
NPs with coronas (post-30 min incubation in BALf) in DI water				
D_H_ (nm)	275 ± 1.6	115 ± 2.1		
PdI	0.43 ± 0.028	0.40 ± 0.053		
Conductance (mS/cm)	0.171 ± 0.006	0.235 ± 0.02		
ζ(mV)	−46.9 ± 1.82	−56.35 ± 3.32		

*D*_*H*_ hydrodynamic diameter

*PdI* polydispersity index

*ζ* zeta potential

*DI water* deionized water

*BALf* bronchoalveolar lavage fluid

**Fig. 5:**
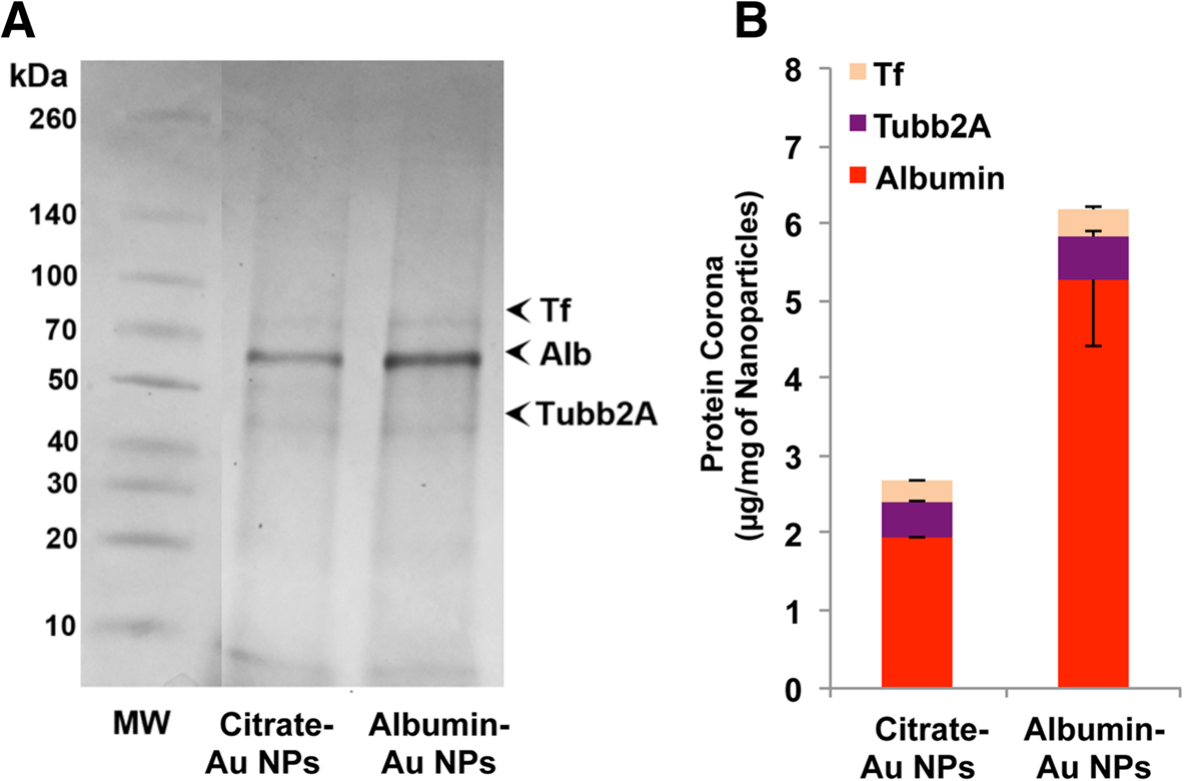
Analysis of Au NPs after incubation with BAL fluid. **a** NP-bound rat BAL proteins were analyzed by 1D gel electrophoresis and mass spectrometry. The molecular weights (kDa) of reference proteins are shown in lane MW. A representative gel from one experiment is shown. Three proteins identified by LC-MS are indicated on right. **b** Quantification of albumin, Tf and Tubb2A adsorbed on Au NPs. Data are mean ± SE (*n* = 4 rats per NP)

First, we examined the composition of acquired protein corona formed on the two Au NPs after incubation in BALf. We found no significant qualitative or quantitative differences in the corona composition between citrate- and albumin-coated Au NPs (Fig. 5). We found that in both cases, the corona primarily contained albumin.

**Fig. 6:**
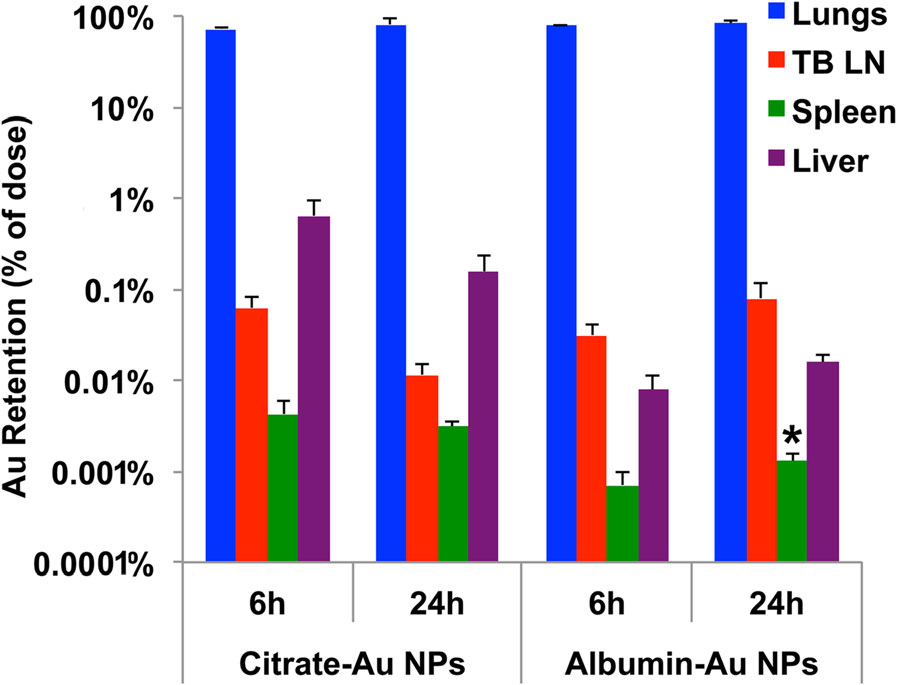
Tissue distribution of Au at 6 and 24 h after IT instillation of citrate- and albumin-coated Au NPs in rats. The bulk of measured Au was found in the lungs. Measured Au levels in the lungs were not different between the two NPs at both time points. Low percentages of instilled Au were measured in the liver, spleen and tracheobronchial lymph nodes. The translocation of Au to the spleen was significantly lower in rats instilled with albumin-coated Au NPs than in rats instilled with citrate-coated Au NPs at 24 h. Data are mean ± SE (*n* = 12 rats per NP). Note: Y-axis in log scale

We then IT-instilled rats with either citrate- or albumin-coated Au NPs and examined the Au content in lungs and other selected organs at 6 and 24 h post-instillation. Our results showed that there was no significant difference in the fate of the two Au NPs (Fig. 6). We examined the Au content in the liver, spleen, the major organs of the mononuclear phagocyte system and tracheobronchial lymph nodes. Extrapulmonary retention of Au from both NPs was in the order liver > lymph node > spleen. The translocation of Au to the spleen was lower in rats instilled with albumin-coated Au NPs (0.001%) than in rats instilled with citrate-coated Au NPs (0.003%). The difference was statistically significant at 24 h post-instillation (Fig. 6).

**Fig. 7:**
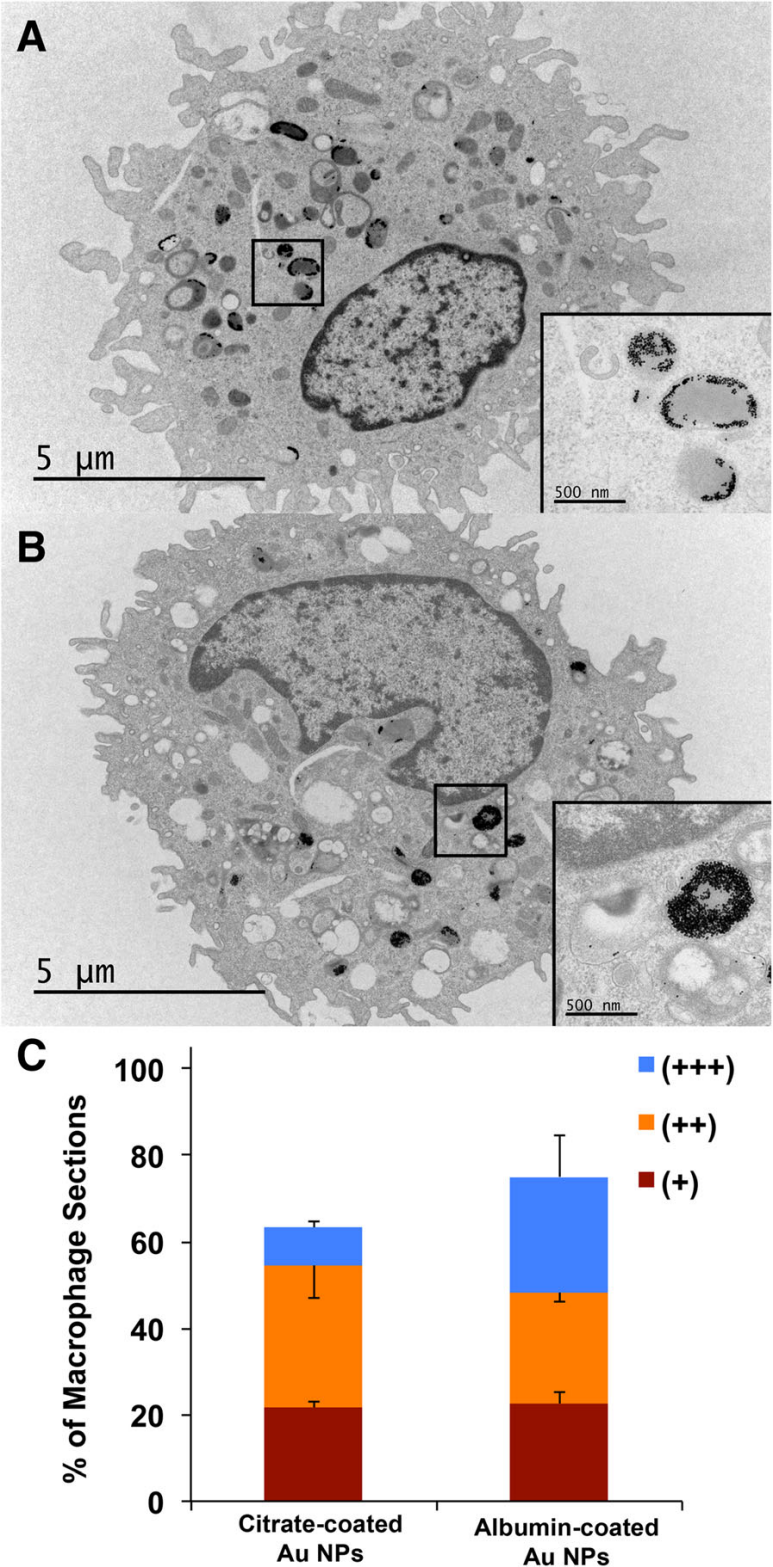
Transmission electron micrographs of lavaged cells at 24 h from rats instilled with Au NP suspensions at a dose of 1 mg/kg body weight. **a** Macrophage uptake of citrate-coated Au NPs and **b** albumin-coated Au NPs. Micrographs in 6A and 6B insets are higher magnification of the areas shown. **c** Morphometric analysis of NP uptake. Macrophage uptake was scored similarly as described in Fig. 3. No significant difference in macrophage uptake of NPs (*n* = 185, citrate-coated Au NPs, *n* = 191, albumin-coated Au NPs) was found. Data are mean ± SE of % of macrophages (*n* = 3 rats per NP)

Our data showed that the resulting coronas of both Au NPs in vitro were predominantly composed of albumin, transferrin and Tubb2A, and were not different between the two Au NPs (Fig. 5). However, the increase in hydrodynamic size of albumin-coated Au NPs was two-fold compared to ten-fold of the citrate-coated Au NPs (Table 3). The zeta potential significantly changed only with albumin-coated Au NPs (−14 to −56 mV). This can be attributed to additional proteins from BALf. Also, the conductance of the suspending medium increased moderately with albumin-coated compared to citrate-coated Au NPs (2.2 vs. 3.6 fold increase). To test if AM uptake of citrate- and albumin-coated Au NPs were different, we also measured the uptake of these NPs in AMs recovered from rats 24 h after instillation. We found that the percentage of macrophage sections with internalized Au NPs was not different between rats instilled with citrate- and albumin-coated Au NPs (Fig. 7). However, macrophages that have taken up albumin-coated Au NPs had fewer endosomes with densely packed NPs compared to macrophages with citrate-coated Au NPs.

## Discussion

Our goal was to characterize the physicochemical properties of CeO_2,_ Si-CeO_2_ BaSO_4_ and ZnO NPs when suspended in distilled water and after incubation in harvested cell-free rat BALf for 30 min at 37 °C. We focused on the protein components of the NP corona; the analysis of the phospholipid component will be reported elsewhere. CeO_2_, Si-CeO_2_, BaSO_4_ and ZnO NPs were incubated in BALf to determine the formation of the protein corona, and its relation to their agglomerate size and zeta potential. After incubation in BALf, CeO_2_ and ZnO NP zeta potentials changed from positive to negative, and the hydrodynamic diameters and conductance of all four NP suspensions increased. Changes in zeta potential, conductance and agglomerate sizes were likely due to acquisition of protein and phospholipid corona from the BALf [27].

Our analyses showed differences in the amounts of adsorbed proteins per mg of NPs (Fig. 2b) and surface area **(**Fig. 2d). Corona proteins were compared based on NP mass and surface area since normalization based on particle number is complicated because of NP agglomeration in liquid media. Moreover, it is difficult to determine whether these proteins adsorb primarily on agglomerate surfaces or can also penetrate the ‘agglomerates’ and coat individual particles.

Differences in the corona proteins among the four NPs may be due to the differences in NP surface chemistry, charge and hydrophobicity that in turn influence NP-biomolecular interactions. The surface chemistry of nanoparticles influences their agglomeration and the formation of coronas around them. It is thought that NPs may aggregate first and then form a corona around the aggregate, or may form coronas around individual particles first and then aggregate. Both aggregation and corona formation are natural processes that lower the surface energy of NPs [28].

Among the corona proteins, amounts of albumin, transferrin and α-1-antitrypsin were relatively greater in the coronas of BaSO_4_ and ZnO than of CeO_2_ and Si-CeO_2_ NPs (Fig. 2c, d). These proteins are among those that can cross the alveolar-epithelial barrier [29]. Receptor-mediated transport in the alveolar epithelium have been reported for albumin and transferrin [29]. Translocation of organic NPs conjugated with human serum albumin has been demonstrated [5]. However, little is known about the precise role of these biomolecules in pulmonary clearance in vivo.

Alveolar macrophages are key resident phagocytic cells of the lungs that sequester and bioprocess NPs as well as orchestrate inflammatory responses [30, 31]. Once internalized in AMs, NPs are slowly cleared from the lungs by particle dissolution and cell migration. The rate at which AMs phagocytize NPs can be influenced significantly by the composition of the corona [17]. Coating magnetite and TiO_2_ with SP-A increases their uptake in macrophages suggesting that the NP protein corona can affect particle recognition, phagocytosis, and processing by AMs [32].

We studied AM uptake of CeO_2_, Si-CeO_2_, BaSO_4_ and ZnO NPs in vivo using transmission electron microscopy (TEM) on lavaged lung cells at 24 h post-instillation. As shown in Fig. 3, the % of AM sections with internalized BaSO_4_ NPs was less than that of CeO_2_ and Si-CeO_2_ NPs. Variations in macrophage uptake of NPs may have been influenced by their surface characteristics and the resulting corona. There were no identifiable ZnO NPs. Even at 1-h post-instillation, ZnO NPs were not observed in lavaged cells (data not shown). The absence of recognizable ZnO NPs in lavaged cells suggests both rapid ingestion by macrophages and fast dissolution due to greater solubility [25]. It is also possible that the release of Zn ions inhibits further ZnO NP uptake by macrophages in vivo.

Our data on proteins in particle coronas showed that transferrin, albumin and A1AT varied significantly among the four NPs. We tested the influence of albumin coating on the fate of instilled Au NPs since it was the major protein observed in all NP coronas. Because Au particles are highly insoluble, we chose them to study the role of albumin in NP translocation from the lungs. In addition, Au NPs can be surface functionalized with organic ligands. Although initially coated with citrate or albumin, Au NPs would likely acquire coronas as they interacted with the lung lining fluid in vivo. Therefore, we examined their protein coronas after incubation in BALf. As shown in Fig. 5, the coronas of both Au NPs predominantly consisted of albumin. The extent to which the apparent higher amount of albumin in the albumin-coated NPs included the original albumin coating could not be determined. Our pharmacokinetic data showed no significant difference in clearance of the two Au NPs from the lungs. Only 0.1 to 0.4% Au NPs translocated from the lungs to extrapulmonary organs. This was consistent with minimal translocation observed in previous studies [19, 33, 34]. Of all organs examined, Au retention in the spleen at 24 h was the only significant difference. It was lower in rats instilled with albumin-coated Au NPs (0.001%) than with citrate-coated Au NPs (0.003%). This small but statistically significant difference in spleen retention needs further investigation.

Our data showed that even with initial coating of citrate or albumin of Au NPs, the resulting coronas formed when incubated in vitro did not differ. They were mainly composed of albumin, transferrin and Tubb2A. The increases in conductance of Au NP suspensions after corona acquisition were similar. However, the increase in hydrodynamic size of albumin-coated Au NPs after incubation in BALf was two-fold compared to ten-fold of the citrate-coated Au NPs (Table 3). The zeta potential changed significantly only with albumin-coated Au NPs (−14 mV to −56 mV). This change in zeta potential might be related to the smaller increase in agglomerate size of albumin-coated Au NPs after acquisition of corona. Hydrodynamic diameters were greater likely due to NP agglomeration, acquisition of protein and phospholipid corona or both. The changes in hydrodynamic diameters of NPs may be explained by competing reactions of NPs with ions that promote agglomeration and with proteins and lipids forming the corona that prevent agglomeration. The total amounts of recovered corona proteins from Au NPs were not significantly different.

We explored if the observed changes in agglomerate sizes and zeta potentials when Au NPs interact with lung lining fluid in vivo also affect their uptake by AMs. The fraction of macrophage sections with internalized Au NPs was about 2.5 times higher than with CeO_2_ NPs (Figs. 3 and 7) suggesting that the corona with more albumin might promote phagocytosis, as shown previously [35]. However, fewer macrophage sections contained internalized BaSO_4_ NPs although their corona had equivalent amounts of albumin compared to that of citrate-coated Au NPs. The difference might be due to greater dissolution of BaSO_4_ than Au NPs in phagolysosomes during the 24-h post-instillation period.

As we found that both the protein corona and the lung clearance of the two Au NPs were not different, it let us consider the corona formation on functionalized or surface-modified NPs that occurs in in vivo experiments. Additional studies are needed to explore the potential role of other corona proteins as well as phospholipids in the fate of NPs in the lungs and in extrapulmonary sites. We hypothesize that the interaction of NPs with biomolecules of the lung lining fluid at the air-blood barrier leads to the formation of an NP corona, which may affect their uptake by AMs and other cells as well as their translocation across the air-blood barrier.

## Conclusions

Our data show that different NPs bind significantly different proteins as they interact with lung lining fluids. Furthermore, we showed that gold NPs coated with citrate or albumin acquired similar protein coronas, were cleared from the lungs, and were retained in extrapulmonary organs to the same degree. The extent to which the protein corona is a critical determinant of the fate and biological effects of NPs remains to be established. Studies examining the independent and collective effects of the major proteins and phospholipids of lung lining fluid forming the NP corona, and their role in regulating translocation of NPs across the air blood barrier and in determining which cells bind and/or ingest NPs, are warranted. We believe that an extensive database of both protein and phospholipid coronas of a diverse NP library will help predict the pulmonary effects and biokinetics of inhaled NPs.

## Methods

### Nanoparticle synthesis and characterization

#### Synthesis of nanoparticles

Uncoated CeO_2_, Si-CeO_2_ and ZnO NPs were made by flame spray pyrolysis using the Versatile Engineered Nanomaterial Generation System (VENGES) at Harvard University [36]. Detailed physicochemical and morphological characterization of these NPs was reported earlier [37, 38]. BaSO_4_ NPs (NM-220) were obtained from BASF SE (Ludwigshafen, Germany). It was a reference material for the Nanomaterial Testing Sponsorship Program of the Organization for Economic Cooperation and Development (OECD). The characterization of the original batch distributed as NM-220 was published recently [39].

Monodisperse Au NPs were prepared with modifications of a previously published protocol [40]. Au NPs were produced by mixing 5.3 mg of NaAuCl_4 *_ 2H_2_O dissolved in 25 mL of Milli-Q grade water with 1 mL of a 1% trisodium citrate solution. Then glass beads were added for mixing and heat distribution. The mixture was boiled in a microwave for 90 s. The deep-red suspension was cooled down slowly to room temperature and stored protected from light at room temperature. An aliquot of prepared Au NPs were functionalized with human serum albumin (HSA) with few modifications from a previously described method [41]. Briefly, 1 mL of colloidal Au NPs at 5 nM was added drop-wise under constant stirring to 500 mL of HSA solutions at 5–10 μM. After incubation for 40 min at room temperature in the dark, the solution was centrifuged for 6 min at 14,000 rpm. The supernatant was removed, and the NPs were washed twice by centrifugation/resuspension in Milli-Q water and finally dispersed in a PBS solution. The size, shape and monodispersity of the NPs were verified by transmission electron microscopy. UV-Vis measurements were performed with an Ultro spec 2100 Pro (Amersham). The maximum absorption of Au NPs is centered at 520 nm (without HSA) and at 525 nm (with HSA). The hydrodynamic diameter (DH), polydispersity index (PdI), and zeta potential (ζ) of each Au-NP suspended in DI water were measured by DLS using a Zetasizer Nano-ZS (Malvern Instruments, Worcestershire, UK). The DLS measurements were performed in ion-free conditions since the citrate-coated Au NPs aggregated in PBS. However, the stability of the AuNPs in ionic solutions increased once the NPs were coated with either albumin alone (data not shown) or after acquisition of protein corona.

#### Hydrodynamic diameter and zeta potential

CeO_2_, silica-coated CeO_2_, BaSO_4_ and ZnO NPs suspensions at specified concentrations in sterile distilled water were sonicated in conical polyethylene tubes. A critical dispersion sonication energy (DSE_cr_) to achieve the smallest particle agglomerate size was used, as previously reported [37]. The suspensions were sonicated at 242 J/ml (20 min/ml at 0.2 watt power output) in a cup sonicator fitted on a Sonifier S-450A (Branson Ultrasonics, Danbury, CT). The sample tubes were immersed in running cold water to minimize heating of the particles during sonication. The hydrodynamic diameter (DH), polydispersity index (PdI), and zeta potential (ζ) of each suspension were measured by DLS using a Zetasizer Nano-ZS (Malvern Instruments, Worcestershire, UK). The Au NP suspensions were similarly analyzed without prior sonication.

#### Characterization of NPs after incubation in BALf: Protein corona and dynamic light scattering analysis

A total of 9 ml pooled BAL fluid from 3 rats (3 ml/rat) was centrifuged at 350 x g to remove cells. Then, CeO_2_, Si-CeO_2_, BaSO_4_ and ZnO (200 μL of 1 mg/mL) were incubated in 3 mL BALf for 30 min at 37 °C. We chose 30-min incubation since corona formation in the lungs occurs soon after the particles interact with the alveolar lining, and because those early events are relevant to their fate in the lungs [42, 43].

After incubation, the NP suspensions were centrifuged for 10 min at 14,500 x g. The resulting pellet was washed in DI water three times. The use of DI water avoided the potential interaction of hydrophobic phospholipid components of the BALf with the corona proteins. The pellets containing NPs with ‘hard corona’ were suspended in 20 μL of DI water. One set was used for DLS analyses and the other set was used for corona protein composition analyses.

The NP pellets for corona protein analyses were also suspended in 20 μL of DI water to which 10 μL of 4× Laemmli sample buffer was added and vortexed. The sample was then heated to 95 °C for 7 min. After cooling to room temperature, 6 μL of mixed solution (57 μL Laemmli and 3 μL β-mercaptoethanol was added to 18 μL of the sample. The samples were then loaded onto a gel and proteins were visualized by 1D SDS-PAGE in combination with Coomassie staining. The protein corona experiment was repeated three times (total of 3 rats/NP) for CeO_2_, Si-CeO_2_, BaSO_4_ and ZnO, and two times for citrate-coated Au and albumin-coated Au NPs (total of 4 rats/NP).

Distinct bands from 2 gels were excised and subjected to a modified in-gel trypsin digestion procedure [44]. Peptides were later extracted and then dried in a Speed-Vac (~1 h). The samples were then stored at 4 °C until analysis. Samples were analyzed at the Harvard Medical School Taplin mass spectrometry facility (Boston, MA). On the day of analysis, the samples were reconstituted in 5–10 μL of HPLC solvent A (2.5% acetonitrile, 0.1% formic acid). A gradient was formed and peptides were eluted with increasing concentrations of solvent B (97.5% acetonitrile, 0.1% formic acid) [45]. Eluted peptides were subjected to electrospray ionization and then analyzed in an LTQ Orbitrap Velos Pro ion-trap mass spectrometer (Thermo Fisher Scientific, San Jose, CA). Peptides were detected, isolated, and fragmented to produce a tandem mass spectrum of specific fragment ions for each peptide. Peptide sequences (and protein identity) were determined by matching protein databases with the acquired fragmentation pattern by the software program, Sequest (ThermoFisher, San Jose, CA) [46]. Spectral matches were manually examined and multiple identified peptides per protein were required. The relative amounts of identified proteins were calculated based gel band densities obtained with ChemiDoc™ XRS System (BioRad, Hercules, CA) and analyzed with ImageJ (NIH).

### Animals

The protocols in this study were approved by the Harvard Medical Area Animal Care and Use Committee (Boston, MA). Fifty two male Wistar Han rats (8 weeks old) were obtained from Charles River Laboratories (Wilmington, MA) and were housed in standard microisolator cages under controlled conditions of temperature, humidity, and light at the Harvard Center for Comparative Medicine. The rats were fed commercial chow (PicoLab Rodent Diet 5053, Framingham, MA) and provided with reverse-osmosis purified water ad libitum. The animals were acclimatized in the facility for 7 days before the start of each experiment. These animals were used for the experiments described below.

### Assessment of uptake of NPs in vivo by alveolar macrophages

CeO_2_, Si-CeO_2_, BaSO_4_, ZnO, citrate-coated Au and albumin-coated Au NP suspensions were instilled in separate cohorts of rats (*n* = 3 per NP) at a dose and concentration of 1 mg/kg and 0.67 mg/ml. At 24 h post-instillation, rats were sacrificed and their lungs were lavaged, as described previously [47]. BAL cells were cytocentrifuged and fixed in 2.5% glutaraldehyde in HEPES buffer, pH 7.4. The pellets were processed for electron microscopy. Uptake of NPs by cells was analyzed in a JEOL 1400 transmission electron microscope (JEOL USA, Inc., Peabody, MA). Random micrographs from each rat were scored for the presence of internalized NPs in each macrophage. Macrophage uptake was scored as +, ++, or +++ when 1–2, 3–4 or ≥5 particle-containing phagosomes were observed in randomly selected electron micrographs, respectively. Alveolar macrophages were scored (CeO_2_ *n* = 248, Si-CeO_2_ *n* = 273, BaSO_4_ *n* = 295, ZnO *n* = 250, citrate-coated Au *n* = 185, albumin-coated Au *n* = 191).

### Pharmacokinetics of Au after intratracheal instillation of Au NPs

Fifty microliters of each NP suspension were added to sterile distilled water to prepare a volume dose of 1.5 ml/kg body weight. Before dosing, each rat was anesthetized with isoflurane (Piramal Healthcare, Bethlehem, PA). Based on ICP-MS analysis, the final dose was 37 μg Au/rat (citrate-coated Au NPs) and 90 μg Au/rat (albumin-coated Au NPs). The NP suspension was intratracheally instilled into the lungs of 12 rats per Au NP. Then, six rats from each group were euthanized at 6 h and 24 h post-dosing. They were anesthetized with vaporized isoflurane, and exsanguinated via the abdominal aorta. The lungs, tracheobronchial lymph nodes, spleen, and liver were collected for Au analysis using ICP-MS. Data were expressed as percentage of the administered dose retained in each organ.

#### Statistical analyses

All data were analyzed using multivariate analysis of variance (MANOVA) followed by Tukey post hoc tests using SAS Statistical Analysis Software (SAS Institute, Cary, NC). The macrophage uptake of Au NPs in vivo was analyzed using Student's t test.

## Abbreviations

A1AT: α1-antitrypsin
AM: alveolar macrophage
Au NPs: gold nanoparticles
BALf: bronchoalveolar lavage fluid
BET: Brunauer–Emmett–Teller
C3: complement component 3
Cfb: complement factor B
D_H_: hydrodynamic diameter
DI: deionized water
DPBS: Dulbecco’s phosphate buffered saline
DSE_cr_: critical dispersion energy
D_xrd_: primary particle size based on X-ray diffraction
ICP-MS: inductively coupled-mass spectrometry
LC-MS: liquid chromatography-mass spectrometry
MW: molecular weight
NP: nanoparticle
PdI: polydispersity index
PL: phospholipid
PLUNC: palate, lung, nasal epithelium clone protein
SDS-PAGE: sodium dodecyl sulfate polyacrylamide gel electrophoresis
SP-A: surfactant protein A
SP-B: surfactant protein B
SP-C: surfactant protein C
SP-D: surfactant protein D
SSA: specific surface area
TEM: transmission electron microscopy
Tf: transferrin
TiO_2_: titanium dioxide
Tubb2A: tubulin Beta 2A Class IIa
UV-vis: ultraviolet-visible spectrophotometry
UV-Vis: visible and ultraviolet spectroscopy
ζ: zeta potential
ρ: density

## References

[1] Martin J, Bello D, Bunker K, Shafer M, Christiani D, Woskie S (2015). Occupational exposure to nanoparticles at commercial photocopy centers. J Hazard Mater.

[2] Pirela SV, Lu X, Miousse I, Sisler JD, Qian Y, Guo N (2016). Effects of intratracheally instilled laser printer-emitted engineered nanoparticles in a mouse model: a case study of toxicological implications from nanomaterials released during consumer use. NanoImpact.

[3] Servin A, White JC (2016). Nanotechnology in agriculture: next steps for understanding engineered nanoparticle exposure and risk. NanoImpact.

[4] Sotiriou GA, Singh D, Zhang F, Chalbot MC, Spielman-Sun E, Hoering L (2016). Thermal decomposition of nano-enabled thermoplastics: possible environmental health and safety implications. J Hazard Mater.

[5] Choi HS, Ashitate Y, Lee JH, Kim SH, Matsui A, Insin N (2010). Rapid translocation of nanoparticles from the lung airspaces to the body. Nat Biotechnol.

[6] Cohen JM, Derk R, Wang L, Godleski J, Kobzik L, Brain J, et al. Tracking translocation of industrially relevant engineered nanomaterials (enms) across alveolar epithelial monolayers in vitro. Nanotoxicology. 2014; 10.3109/17435390.2013.879612 PMC4387897 24479615

[7] Brain JD, Fishman AP, Fishmn AB (1985). Macrophages in the respiratory tract. Handbook of physiology - section 3.

[8] Brain JD (1992). Mechanisms, measurement, and significance of lung macrophage function. Environ Health Perspect.

[9] Bastacky J, Lee CY, Goerke J, Koushafar H, Yager D, Kenaga L (1995). Alveolar lining layer is thin and continuous: low-temperature scanning electron microscopy of rat lung. J Appl Physiol.

[10] Goerke J (1998). Pulmonary surfactant: functions and molecular composition. Biochim Biophys Acta.

[11] Kendall M, Ding P, Mackay RM, Deb R, McKenzie Z, Kendall K (2013). Surfactant protein d (sp-d) alters cellular uptake of particles and nanoparticles. Nanotoxicology.

[12] Pikaar JC, Voorhout WF, van Golde LM, Verhoef J, Van Strijp JA, van Iwaarden JF (1995). Opsonic activities of surfactant proteins a and d in phagocytosis of gram-negative bacteria by alveolar macrophages. J Infect Dis.

[13] Monopoli MP, Aberg C, Salvati A, Dawson KA (2012). Biomolecular coronas provide the biological identity of nanosized materials. Nat Nanotechnol.

[14] Monopoli MP, Walczyk D, Campbell A, Elia G, Lynch I, Bombelli FB (2011). Physical-chemical aspects of protein corona: relevance to in vitro and in vivo biological impacts of nanoparticles. J Am Chem Soc.

[15] Fan Q, Wang YE, Zhao X, Loo JS, Zuo YY (2011). Adverse biophysical effects of hydroxyapatite nanoparticles on natural pulmonary surfactant. ACS Nano.

[16] Hu G, Jiao B, Shi X, Valle RP, Fan Q, Zuo YY (2013). Physicochemical properties of nanoparticles regulate translocation across pulmonary surfactant monolayer and formation of lipoprotein corona. ACS Nano.

[17] Shaw CA, Mortimer GM, Deng ZJ, Carter ES, Connell SP, Miller MR (2016). Protein corona formation in bronchoalveolar fluid enhances diesel exhaust nanoparticle uptake and pro-inflammatory responses in macrophages. Nanotoxicology.

[18] Schaffler M, Semmler-Behnke M, Sarioglu H, Takenaka S, Wenk A, Schleh C (2013). Serum protein identification and quantification of the corona of 5, 15 and 80 nm gold nanoparticles. Nanotechnology.

[19] Schleh C, Holzwarth U, Hirn S, Wenk A, Simonelli F, Schaffler M (2013). Biodistribution of inhaled gold nanoparticles in mice and the influence of surfactant protein d. J Aerosol Med Pulm Drug Deliv.

[20] Ruge CA, Schaefer UF, Herrmann J, Kirch J, Canadas O, Echaide M (2012). The interplay of lung surfactant proteins and lipids assimilates the macrophage clearance of nanoparticles. PLoS One.

[21] Geiser M, Kreyling WG (2010). Deposition and biokinetics of inhaled nanoparticles. Part Fibre Toxicol.

[22] Konduru N, Keller J, Ma-Hock L, Groters S, Landsiedel R, Donaghey TC (2014). Biokinetics and effects of barium sulfate nanoparticles. Part Fibre Toxicol.

[23] Konduru NV, Jimenez RJ, Swami A, Friend S, Castranova V, Demokritou P (2015). Silica coating influences the corona and biokinetics of cerium oxide nanoparticles. Part Fibre Toxicol.

[24] Konduru NV, Murdaugh KM, Sotiriou GA, Donaghey TC, Demokritou P, Brain JD (2014). Bioavailability, distribution and clearance of tracheally-instilled and gavaged uncoated or silica-coated zinc oxide nanoparticles. Part Fibre Toxicol.

[25] Sotiriou GA, Watson C, Murdaugh KM, Darrah TH, Pyrgiotakis G, Elder A (2014). Engineering safer-by-design, transparent, silica-coated zno nanorods with reduced DNA damage potential. Environ Sci Nano.

[26] Moyano DF, Saha K, Prakash G, Yan B, Kong H, Yazdani M (2014). Fabrication of corona-free nanoparticles with tunable hydrophobicity. ACS Nano.

[27] Pyrgiotakis G, Blattmann CO, Demokritou P (2014). Real-time nanoparticle-cell interactions in physiological media by atomic force microscopy. ACS Sustain Chem Eng.

[28] Tsuda A, Konduru NV (2016). The role of natural processes and surface energy of inhaled engineered nanoparticles on aggregation and corona formation. NanoImpact.

[29] Kim KJ, Malik AB (2003). Protein transport across the lung epithelial barrier. Am J Physiol Lung Cell Mol Physiol.

[30] Brain JD, Knudson DE, Sorokin SP, Davis MA (1976). Pulmonary distribution of particles given by intratracheal instillation or by aerosol inhalation. Environ Res.

[31] Geiser M, Casaulta M, Kupferschmid B, Schulz H, Semmler-Behnke M, Kreyling W (2008). The role of macrophages in the clearance of inhaled ultrafine titanium dioxide particles. Am J Respir Cell Mol Biol.

[32] Ruge CA, Kirch J, Canadas O, Schneider M, Perez-Gil J, Schaefer UF (2011). Uptake of nanoparticles by alveolar macrophages is triggered by surfactant protein a. Nanomedicine.

[33] Kreyling WG, Hirn S, Moller W, Schleh C, Wenk A, Celik G (2014). Air-blood barrier translocation of tracheally instilled gold nanoparticles inversely depends on particle size. ACS Nano.

[34] Lipka J, Semmler-Behnke M, Sperling RA, Wenk A, Takenaka S, Schleh C (2010). Biodistribution of peg-modified gold nanoparticles following intratracheal instillation and intravenous injection. Biomaterials.

[35] Vuarchey, C, Kumar, S, R, S: albumin coated liposomes: a novel platform for macrophage specific drug delivery. Nanotechnol Dev 2011, 1: 5–10.

[36] Demokritou P, Buchel R, Molina RM, Deloid GM, Brain JD, Pratsinis SE (2010). Development and characterization of a versatile engineered nanomaterial generation system (venges) suitable for toxicological studies. Inhal Toxicol.

[37] Cohen J, Deloid G, Pyrgiotakis G, Demokritou P (2013). Interactions of engineered nanomaterials in physiological media and implications for *in vitro* dosimetry. Nanotoxicology.

[38] Demokritou P, Gass S, Pyrgiotakis G, Cohen JM, Goldsmith W, McKinney W (2013). An in vivo and in vitro toxicological characterisation of realistic nanoscale ceo(2) inhalation exposures. Nanotoxicology.

[39] Wohlleben W, Ma-Hock L, Boyko V, Cox G, Egenolf H, Freiberger H (2013). Nanospecific guidance in reach: a comparative physical-chemical characterization of 15 materials with methodical correlations. JCeramSciTech.

[40] J., T, C., SP, J., H: A study of the nucleation and growth processes in the synthesis of colloidal gold. Discuss Faradya Soc 1951, 11: 55–75.

[41] Brewer SH, Glomm WR, Johnson MC, Knag MK, Franzen S (2005). Probing bsa binding to citrate-coated gold nanoparticles and surfaces. Langmuir.

[42] Frost R, Langhammer C, Cedervall T (2017). Real-time in situ analysis of biocorona formation and evolution on silica nanoparticles in defined and complex biological environments. Nano.

[43] Khan S, Gupta A, Verma NC, Nandi CK (2015). Kinetics of protein adsorption on gold nanoparticle with variable protein structure and nanoparticle size. J Chem Phys.

[44] Shevchenko A, Wilm M, Vorm O, Mann M (1996). Mass spectrometric sequencing of proteins silver-stained polyacrylamide gels. Anal Chem.

[45] Peng J, Gygi SP (2001). Proteomics: the move to mixtures. J Mass Spectrom.

[46] Eng JK, McCormack AL, Yates JR (1994). An approach to correlate tandem mass spectral data of peptides with amino acid sequences in a protein database. J Am Soc Mass Spectrom.

[47] Beck BD, Brain JD, Bohannon DE (1982). An in vivo hamster bioassay to assess the toxicity of particulates for the lungs. Toxicol Appl Pharmacol.

